# Magnetic structure study of the sawtooth chain antiferromagnet $\hbox {Fe}_2\hbox {Se}_2\hbox {O}_7$

**DOI:** 10.1038/s41598-021-03058-5

**Published:** 2021-12-15

**Authors:** Kazuhiro Nawa, Maxim Avdeev, Peter Berdonosov, Alexey Sobolev, Igor Presniakov, Alena Aslandukova, Ekaterina Kozlyakova, Alexander Vasiliev, Igor Shchetinin, Taku J. Sato

**Affiliations:** 1 Institute of Multidisciplinary Research for Advanced Materials, Tohoku University, 2-1-1 Katahira, Sendai, 980-8577 Japan; 2 Australian Centre for Neutron Research, Australian Nuclear Science and Technology Organisation, Kirrawee DC, NSW 2232 Australia; 3 School of Chemistry, The University of Sydney, Sydney, 2006 Australia; 4 National University of Science and Technology MISIS, Moscow, 119049 Russia; 5 Lomonosov Moscow State University, Moscow, 119991 Russia; 6 Bavarian Research Institute of Experimental Geochemistry and Geophysics, University of Bayreuth, Bayreuth, 95447 Germany

**Keywords:** Condensed-matter physics, Magnetic properties and materials, Quantum fluids and solids

## Abstract

A magnetic structure of the sawtooth-chain antiferromagnet $\hbox {Fe}_2\hbox {Se}_2\hbox {O}_7$ was investigated by magnetization measurements, single crystalline and powder neutron diffraction experiments, and a further analysis on the Mössbauer spectra. These experiments revealed a nearly collinear antiferromagnetic structure with magnetic moments aligned along the *b*-axis, indicating dominant antiferromagnetic exchanges between Fe(1)–Fe(2) and Fe(2)–Fe(3) sites. The magnon dispersion relation derived from the linear spin wave approximation suggests the possible flat band nature of magnons.

## Introduction

A variety of spin chain systems have been investigated so far to search for unconventional phases, excitations, and critical phenomena^1,2^. Among them, spin chains involving competing interactions, such as a spin-1/2 symmetric zigzag spin ladder and a sawtooth (or delta) chain models have attracted interest in terms of topological excitations. The symmetric zigzag spin ladder model represents a spin chain with nearest ($J_1$) and next-nearest neighbor ($J_2$) magnetic interactions. The spin-1/2 symmetric zigzag spin ladder model with $J_1$/$J_2$ = 2 exhibits the twofold-degenerate singlet ground state^3,4^. Topological excitations separate singlet domains and behave as defects propagating along the chain^5,6^. The sawtooth chain model represents a spin chain with corner-sharing triangles forming a one-dimensional chain. The model consists of magnetic interactions at vertex-base ($J_\mathrm {vb}$) and base-base bonds ($J_\mathrm {bb}$). The ground state of the spin-1/2 sawtooth chain model with $J_\mathrm {vb}$/$J_\mathrm {bb}$ = 1 is twofold degenerate^7^ and identical to that of the spin-1/2 symmetric zigzag spin ladder model under periodic boundary conditions^8^. In excited states, a single spin located at the vertex site separates two different types of domains: one consists of triangles with a singlet dimer located at the left side while the other consists of triangles with a dimer located at the right side. The presence of the two inequivalent spin sites modifies the domain-wall like excitations compared with those of the symmetric zigzag spin ladder model, leading to localized *kinks* without excitation energy and delocalized *antikinks* with a finite excitation energy^8–11^.

Even for the classical limit, the sawtooth chain model can possess flat-band magnons. The well-known example is the spin-1/2 sawtooth chain model with $J_\mathrm {vb}$/$J_\mathrm {bb}$ = 2 at high fields^12,13^. One of the two magnon branches becomes flat above the saturation field, reflecting the localized nature within a valley between two adjacent triangles. Below the saturation field, the ground state is replaced by a magnon crystal state, where every second valley is occupied by localized magnons. Due to the high degeneracy realized at the saturation field, the sawtooth system also attracts attention as potential materials for low-temperature magnetic refrigeration^14^.

Several compounds were reported to have a magnetic sawtooth chain such as $\hbox {YCuO}_{2.5}$^15,16^, $\hbox {Cu}_2$($\hbox {AsO}_4$)(OH) $\cdot$ 3$\hbox {H}_2$O^17^, $\hbox {Cu}_2$Cl(OH)$_3$^18^, {[Cu(bpy)($\hbox {H}_2$O)][Cu(bpy)(mal)($\hbox {H}_2$O)]}($\hbox {ClO}_4$)$_2$ (bpy = 2,2’-bipyridine and mal = malonate dianion)^19,20^, $\hbox {ZnLn}_2\hbox {S}_4$ (Ln = Er, Tm, Yb)^21^ and $\hbox {Rb}_2\hbox {Fe}_2$O($\hbox {AsO}_4$)$_2$^22^. In this article, we discuss magnetic properties of a sawtooth chain magnet $\hbox {Fe}_2\hbox {Se}_2\hbox {O}_7$^23–25^. This compound crystallizes in the space group *Pccn* (*Z* = 8). The Curie-Weiss fit to the temperature dependence of the magnetic susceptibility yields Weiss temperature of −200(10) K, indicating predominant antiferromagnetic interactions^25^. As the temperature is decreased below 300 K, the magnetic susceptibility deviates from the Curie-Weiss rule and shows a shoulder around 120 K^24,25^. The short range order should develop above the transition temperature due to the low-dimensionality in the magnetism. The antiferromagnetic order occurs at 105 K, followed by a sharp increase in the magnetic susceptibility^24,25^ and the increase in the distribution of the hyperfine fields observed by the $^{57}$Fe Mössbauer spectroscopy^25^. In addition, the excitations associated with the magnetic order have been detected by the Raman scattering study^24^. However, it is not so clear whether $\hbox {Fe}_2\hbox {Se}_2\hbox {O}_7$ can be regarded as a sawtooth chain antiferromagnet due to its complicated crystal structure. To confirm that a sawtooth antiferromagnetic chain is formed in $\hbox {Fe}_2\hbox {Se}_2\hbox {O}_7$, we performed magnetization measurements, the single crystalline and powder neutron diffraction experiments, and reanalyzed the Mössbauer spectra. The magnetic structure revealed from these experiments is consistent with the formation of the sawtooth antiferromagnetic chain along the *a*-axis.

## Results

### Magnetization

**Figure 1:**
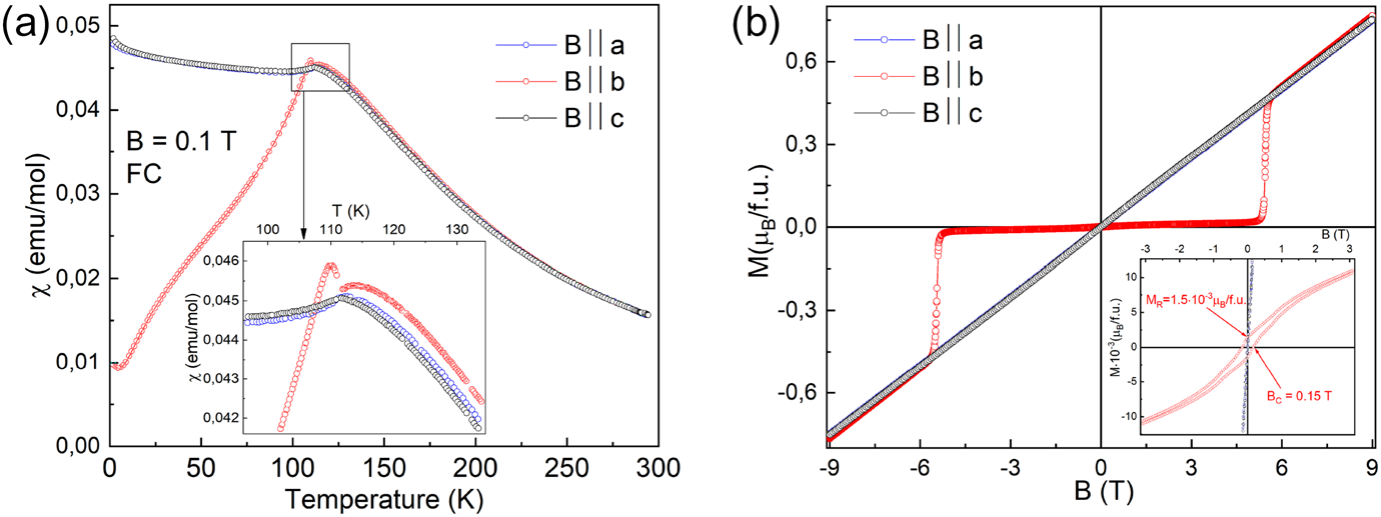
(**a**) Temperature dependence of the magnetic susceptibility measured under the magnetic field of 0.1 T along the *a*, *b*, and *c* axes. The inset shows the expanded view around the magnetic transition. (**b**) Magnetization curve at 2 K. The inset shows the expanded view for the magnetization at low fields.

An easy-axis direction is found from the anisotropy in the magnetization below the transition temperature. The temperature dependence of the magnetic susceptibility for $B \parallel a, b, c$ is shown in Fig. 1a. Below 112 K, the magnetic susceptibility for $B \parallel b$ shows a small hump, and start to decrease down to the base temperature. On the other hand, the magnetic susceptibility for $B \parallel a, c$ does not change so much with decreasing temperature. The anisotropy in the magnetic susceptibility indicates that the *b*-axis is the easy axis of the magnetization. The magnetic anisotropy is supported by the magnetization curve shown in Fig. 1b. The jump in the magnetization was found for $B \parallel b$ at $B_\mathrm {sf}$ = 5.45 T, which should correspond to a spin flop transition. The small magnetization below $B_\mathrm {sf}$ suggests that the magnetic moments are almost aligned along the *b*-axis. The magnetization along the *b*-axis includes a weak ferromagnetic component of $1.5 \times 10^{-3} \mu _\mathrm {B}$, as shown in the inset of Fig. 1b.

**Figure 2:**
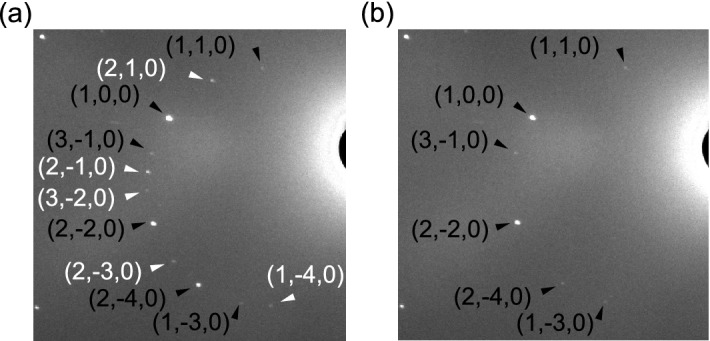
Laue diffraction patterns collected at (**a**) 4 K and (**b**) 120 K together with indices. The wavelength range is restricted to 0.85–1.7 Å for indexing. White color indicates magnetic reflections that are only present below $T_\mathrm {N}$. The contrast of the images are adjusted using the scikit-image library^26^.

**Figure 3:**
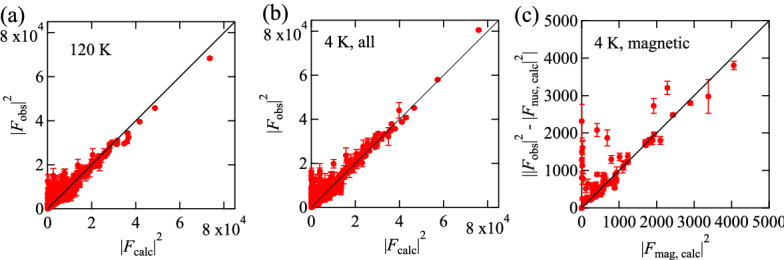
$|F_\mathrm {obs}|^2$ vs $|F_\mathrm {calc}|^2$ plot for (**a**) 120 K and (**b**) 4 K. (**c**) The magnetic contributions $||F_\mathrm {obs}|^2 - |F_\mathrm {nuc, calc}|^2|$ plotted as a function of the calculated squared magnetic structure factor $|F_\mathrm {calc, mag}|^2$ at 4 K. The plot only includes the reflections that satisfy $||F_\mathrm {obs}|^2 - |F_\mathrm {nuc, calc}|^2| > 3\sigma$ and $I(4~\mathrm {K}) - I(120~\mathrm {K}) > 3 (\sigma (4~\mathrm {K})^2 + \sigma (120~\mathrm {K})^2)^{1/2}$. The refinement parameters are listed in Table S1.

### Neutron diffraction

The crystal structure found from the neutron Laue diffraction data is consistent with that determined from the single crystalline X-ray diffraction experiments^23^. The Laue diffraction patterns collected at 120 and 4 K are compared in Fig. 2a,b, respectively. At 120 K the observed peaks are indexed by the space group *Pccn*. For all the temperatures, the refinement yields good agreement between $|F_\mathrm {obs}|^2$ and $|F_\mathrm {calc}|^2$, as shown in Fig. 3a,b and Fig. S1a–e. (The detail of the magnetic structure analysis is discussed later.) The crystallographic data and the refinement parameters are listed in Table S1. The atomic positions at each temperature determined from the refinement are listed in Table S2–S6. The refinement of the neutron powder diffraction experiments also yields the crystal structure consistent with that found from the neutron Laue diffraction data. Crystallographic data and refinement parameters of the powder neutron diffraction experiments at different temperatures are summarized at Table S7. The atomic positions are listed in Table S8–S11.

**Figure 4:**
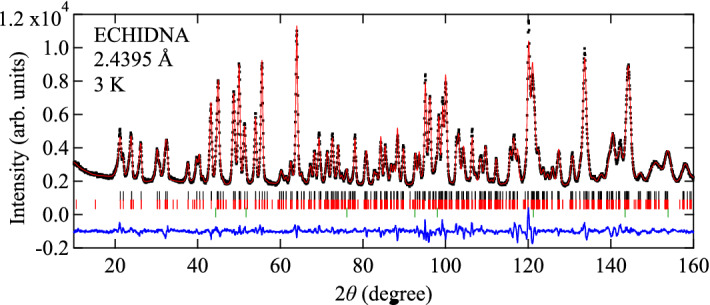
Powder neutron diffraction patterns at 3 K collected with the wavevector of 2.4395 Å together with the Rietveld analysis. Observed, calculated, and the difference between both intensities are shown by black dots, red, and blue curves, respectively. Reflection positions from the main phase (nuclear), the main phase (magnetic), and the secondary phase (NaCl) are indicated by black, red, and green vertical lines, respectively. The weight fraction of the secondary phase is estimated as 1.46(4) wt% from the refinement.

**Figure 5:**
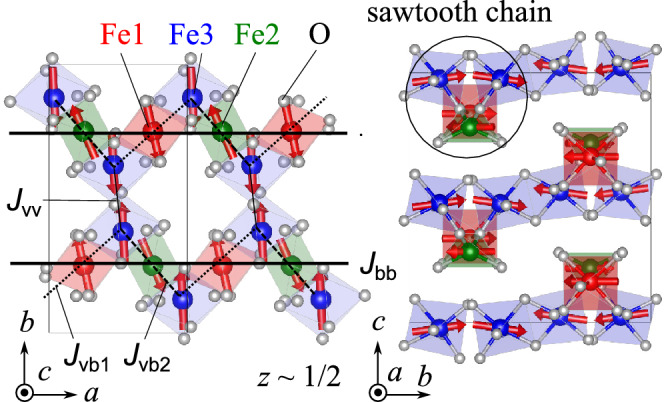
Crystal and magnetic structure in $\hbox {Fe}_2\hbox {Se}_2\hbox {O}_7$ found from the neutron diffraction experiments. The thick solid, dashed, dotted, and thin solid lines indicate the dominant exchange at Fe(1)–Fe(2) ($J_\mathrm {bb}$), Fe(2)–Fe(3) ($J_\mathrm {vb2}$), Fe(1)–Fe(3) ($J_\mathrm {vb1}$), and Fe(3)–Fe(3) ($J_\mathrm {vv}$) bonds indicated by the DFT calculations, respectively. The rectangles indicate the unit cell. The figure is illustrated using VESTA software (Ver.3.5.7, https://jp-minerals.org/vesta/en/)^27^.

**Figure 6:**
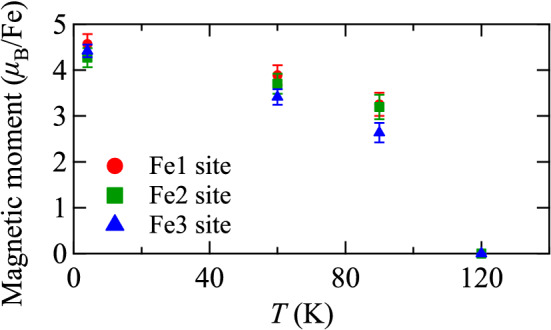
Temperature dependence of the magnitude of the magnetic moment at Fe(1), Fe(2), and Fe(3) sites based on the single crystal neutron diffraction data.

As the temperature is lowered, magnetic reflections appear due to the occurrence of the magnetic order. The reflections such as (1,−4,0), (2,−3,0), (2,−1,0), (2,1,0), and (3,−2,0) are observed at 4 K, while they are not present at 120 K. This is reasonable due to the extinction rule of (*h*, *k*, 0) reflections ($h + k = 2n$, *n* : integer) under the space group of *Pccn*. Other magnetic reflections overlap with nuclear reflections. This suggests the development of the magnetic order with the propagation vector ${\mathbf {q}}$ = 0. The propagation vector is also consistent with the powder neutron diffraction patterns shown in Fig. S2. To determine the magnetic structure through the refinement, candidates for initial magnetic structures are obtained using magnetic representation theory^28^. The calculations were carried out using BasIreps^29^ software. $\hbox {Fe}_2\hbox {Se}_2\hbox {O}_7$ includes three inequivalent Fe sites in a unit cell as shown in Table S12. Fe(1), Fe(2), Fe(3) sites occupy Wyckoff positions 4c, 4d, and 8e, respectively. Fe(1) and Fe(2) sites correspond to the basal sites of the sawtooth chain, while Fe(3) site corresponds to the apical site. Magnetic representations for the Fe moments are decomposed using the irreducible representations (IR) of the k-group with *k* = (0,0,0), which is the same as the original space group *Pccn*. The result of the decomposition is

1 $$\begin{aligned}&\Gamma _\mathrm {(Fe(1)/Fe(2))} ~= \Gamma _1 + \Gamma _2 + \Gamma _3 + \Gamma _4 + 2\Gamma _5 + 2\Gamma _6 + 2\Gamma _7 + 2\Gamma _8 \end{aligned}$$

2 $$\begin{aligned}&\Gamma _\mathrm {(Fe(3))} ~= 3\Gamma _1 + 3\Gamma _2 + 3\Gamma _3 + 3\Gamma _4 + 3\Gamma _5 + 3\Gamma _6 + 3\Gamma _7 + 3\Gamma _8 \end{aligned}$$

and corresponding magnetic basis vectors (BVs) for all the IRs are obtained, as listed in Table S13. We looked for a possible magnetic structure compatible with both the integrated intensities in the single crystal neutron Laue diffraction pattern and the peak intensities in the powder diffraction pattern. The intensities collected at 4 K are reproduced by the $\Gamma _8$ representation for all the three Fe sites, which can be also represented by the magnetic space group $Pc^\prime c n$ (#56.367). The refinement on the integrated intensities in the single crystal neutron Laue diffraction data yields the coefficients for the BVs to be $C_{\mathrm {Fe(1)}, a}$  = −0.94(12), $C_{\mathrm {Fe(1)}, b}$  = 4.47(10), $C_{\mathrm {Fe(2)}, a}$  = 1.60(11), $C_{\mathrm {Fe(2)}, b}$  = 3.96(10), $C_{\mathrm {Fe(3)}, a}$  = −0.22(6), $C_{\mathrm {Fe(3)}, b}$  = −4.37(8), $C_{\mathrm {Fe(3)}, c}$  = 0.6(2) $\mu _B$. The coefficient $C_{i, j}$ represents the *j*-component at the Fe(*i*) site, which is equal to the projection of the magnetic moment along the *j*-direction. $C_{\mathrm {Fe(1)},c}$ and $C_{\mathrm {Fe(2)},c}$ are required to be 0 by the symmetry. The magnetic moments at Fe(1), Fe(2), and Fe(3) sites are almost aligned along the *b*-axis. Their canting angles from the *b*-axis are 11.9(15), 22.0(15), and 8.3(20) degrees at 4 K. The magnetic contributions are extracted by subtracting the nuclear contributions from the total intensity ($||F_\mathrm {obs}|^2 - |F_\mathrm {nuc, calc}|^2|$), and plotted against the calculated squared magnetic structure factor $|F_\mathrm {calc, mag}|^2$in Fig. 3c. The agreement supports the magnetic structure found from the refinement. The magnetic structure also well explains the powder diffraction pattern at 4 K. The powder diffraction pattern is well fitted by fixing the moment direction and only adjusting the moment size, as shown in Fig. 4. Note that the origin of the weak ferromagnetic moment along the *b*-axis has not been understood so far since it is not allowed in the $\Gamma _8$ representation. The weak ferromagnetic component does not affect our analysis at all since its magnitude is smaller than 10$^{-3}$ of the magnetic moment.

The spin model and the refined magnetic structure including two coupled sawtooth chains are illustrated in Fig. 5. For all the Fe sites, the magnetic moments are almost aligned along the *b*-direction. A nearest-neighbor pair of magnetic moments at Fe(1) and Fe(2) sites indicates roughly opposite directions, forming antiferromagnetic chains along the *a*-axis. In addition, nearest-neighbor pairs of magnetic moments at Fe(2) and Fe(3) sites are also aligned in an antiparallel manner. A pair of sawtooth chains is antiferromagnetically coupled along the *b*-axis.

The magnetic structure refinement is also applied to the powder and single crystal neutron diffraction patterns collected at 60 and 90 K. The $|F_\mathrm {obs}|^2$ vs $|F_\mathrm {calc}|^2$ plot for 60 and 90 K is shown in Fig. S1b,c, respectively. Estimated parameters are listed in Table S1 and the temperature dependence of the moment size is shown in Fig. 6. The magnitude of the magnetic moment is consistent with those found in the Mössbauer experiments^25^.

### Mössbauer spectroscopy

**Figure 7:**
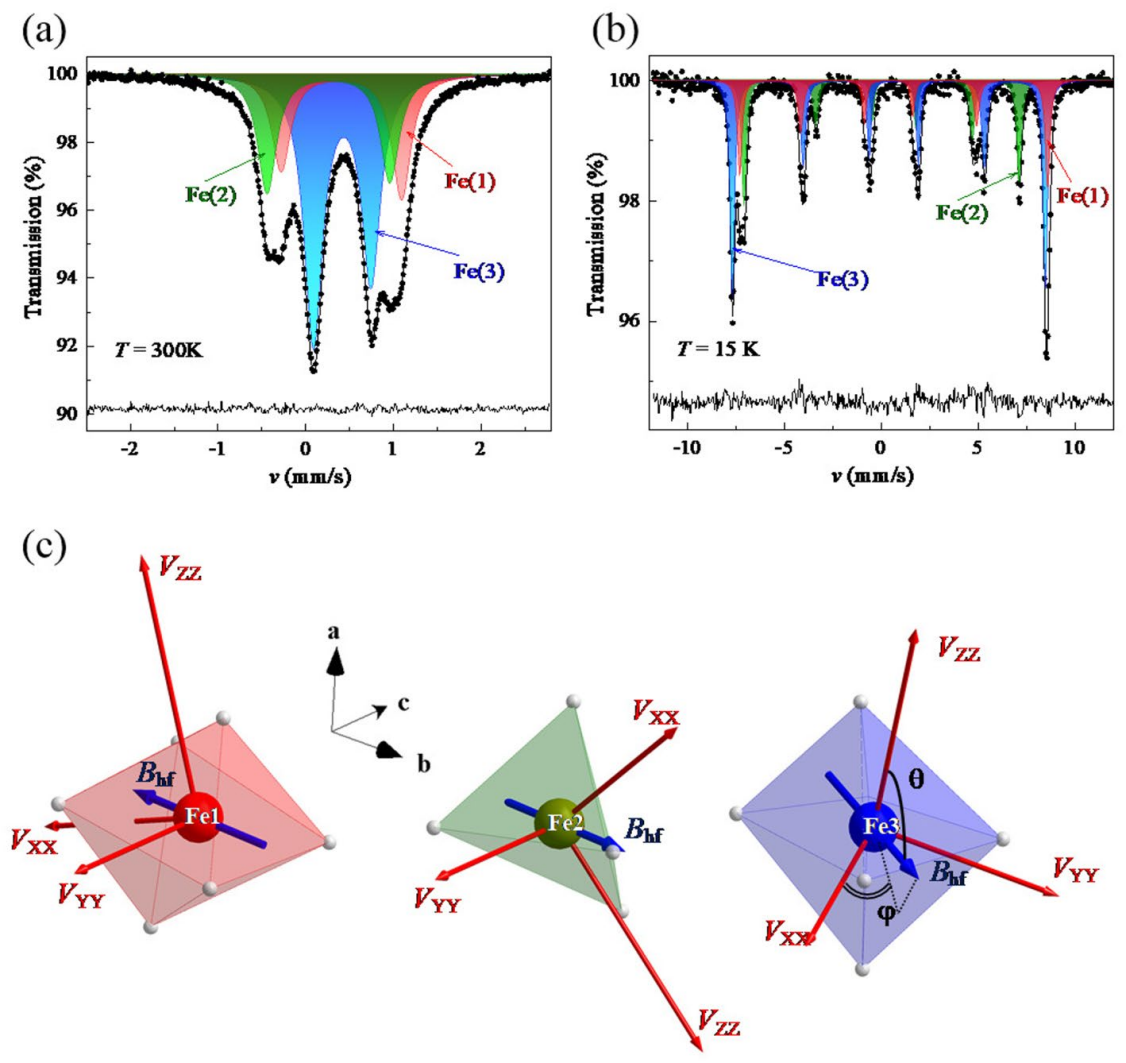
$^{57}$Fe Mössbauer spectra (experimental hollow dots) recorded at (**a**) 300 K (above $T_\mathrm {N}$) and (**b**) 15 K (below $T_\mathrm {N}$)^25^. Solid lines are simulation of the experimental spectra as described in the text. (**c**) Directions of the electric field gradient (EFG) tensor principal axes ($V_{ii}$) and the hyperfine magnetic field ($B_\mathrm {hf}$) for Fe(1), Fe(2), and Fe(3) sites. The atomic coordinate of the Fe atoms shown in the Figure corresponds to(0.75, 0.75, 0.66383), (0.75, 0.25, 0.28279) and (0.53556, 0.12435, 0.97663) for Fe(1), Fe(2), and Fe(3) sites, respectively. Note that the direction of $V_{YY}$ at Fe(1) and Fe(2) sites is exactly along the *c*-direction because of the twofold rotation around the *c*-axis.

The direction of the magnetic moments was also confirmed from the further analysis on the Mössbauer spectra. The Mössbauer spectra at 300 K (above $T_\mathrm {N}$) and 15 K (below $T_\mathrm {N}$) are shown in Fig. 7a,b, respectively. The spectra at the paramagnetic phase is assigned as the three quadrupole doublets Fe(1), Fe(2) and Fe(3) due to the quadrupole splitting of the excited state. The calculated total electric field gradient (EFG) tensor based on the monopole and dipole model as well as the “overlap” contribution were used for the assignment^25^.

When the sample was cooled down to 15 K, three well-resolved sextets appeared in the spectrum due to the additional Zeeman splitting. Since the quadrupole and Zeeman splittings are comparable in the magnitude, the spectra were analyzed using the full Hamiltonian ($H_Q$) including magnetic and quadrupole interactions^30^:

3 $$\begin{aligned} H_\mathrm {Q} = \frac{eQV_{ZZ}}{4I(2I-1)} [3I_Z^2 - I^2 + \eta (I^2_X - I^2_Y)] - g \mu _\mathrm {N} B_\mathrm {hf} [(I_X \cos \psi + I_Y \sin \psi ) \sin \theta + I_Z \cos \theta ], \end{aligned}$$

where *eQ*, *I*, and $I_{X,Y,Z}$ are the $^{57}$Fe nucleus quadrupole moment^31^, the nuclear spin, and nuclear spin projection operators onto the principal axes; $\theta$ and $\psi$ are angular and polar angles of the magnetic hyperfine field $B_\mathrm {hf}$ in the EFG coordinate system. The $H_Q$ eigenvalues depend not only on the hyperfine parameters of the system ($\delta$, $eQV_{ZZ}$, $B_\mathrm {hf}$, and $\eta$) but also on the direction of the hyperfine field, $\theta$ and $\psi$. The eigenvalues of the full Hamiltonian of the combined electrical and magnetic interactions were found by fixing $eQV_{ZZ}$ and $\eta$ to the values calculated from the monopole-dipole model with inclusion of the overlapping contribution^25^. The calculation based on the crystal structure found by the neutron Laue experiments at 4 K is summarized in Table S14. In addition, the orientations of the principal axes relative to the crystallographic axis for each Fe site are illustrated in Fig. 7c. It should be noted that several crystallographic equivalent Fe sites are present in the unit cell, which have the different orientations of the EFG principal axes due to the glide symmetry. The magnitude of the EFG tensor is almost the same as that estimated for the room temperature structure so that hyperfine parameters completely coincide with the previously obtained values^25^.

The direction of the magnetic moments can be estimated from the polar angles ($\theta _i$, $\psi _i$) defined against the principal axes of the EFG tensor. The magnetic moment should be antiparallel to the hyperfine field ($\mu _\mathrm {Fe} \parallel B_\mathrm {hf}$) for the high-spin $\hbox {Fe}^{3+}$ cations, since the contribution of the orbital angular moment should be negligible and thus the hyperfine tensor is isotropic. The directions of the hyperfine fields are illustrated in Fig. 7c. The magnetic moments $\mu _\mathrm {Fe(1)}$ and $\mu _\mathrm {Fe(2)}$ are almost aligned along the *b*-axis (the canting angles are 4$^\circ$ and  2.5$^\circ$, respectively), while the magnetic moments $\mu _\mathrm {Fe(3)}$ are 23$^\circ$ canted from the *b*-direction inside the *bc*-plane. The estimation of the hyperfine field direction includes the ambiguity that the polar angle of ($\theta _i$, $\psi _i$) cannot be distinguished from ($\theta _i \pm \pi$, $\psi _i$) and ($\theta _i$, $\psi _i \pm \pi$) from the Mössbauer spectra. However, the above almost collinear structure agrees well with the magnetic structure determined from the neutron scattering experiments, supporting the validity of our solution. The polar angles ($\theta _i$, $\psi _i$) do not depend on temperature in the whole magnetic ordering region (see Fig. S3), indicating the stability of the full Hamiltonian of combined hyperfine interactions.

## Discussion

The magnetic structure indicates that the antiferromagnetic interactions between Fe(1) and Fe(2) sites and Fe(2) and Fe(3) sites dominate the magnetic structure, which is consistent with the expectation from the crystal structure^25^. The magnetic exchange between the nearest Fe–Fe bonds should be strongly influenced by the Fe–O–Fe bond angle according to the Goodenough-Kanamori rule. Let us discuss the bond angles based on the room-temperature structure found from the single crystal neutron diffraction experiments. The Fe(2)$\hbox {O}_4$ tetrahedra share one oxygen atom with the nearest Fe(1)$\hbox {O}_6$ and Fe(3)$\hbox {O}_6$ octahedra, resulting in the bond angle of 127.8$^\circ$ (Fe(1)–Fe(2)) and 123.9 (Fe(2)–Fe(3)). On the other hand, the Fe(1)$\hbox {O}_6$ and Fe(3)$\hbox {O}_6$ octahedra, or two Fe(3)$\hbox {O}_6$ octahedra are linked in the edge-shared geometry, leading to the much smaller bond angles of 93.6 and 107.7 (Fe(1)–Fe(3)), 104.3 (Fe(3)–Fe(3), the two bonds have the same bond angle). Thus, in terms of the Fe–O–Fe bond angle, it is natural that antiferromagnetic interactions between Fe(1) and Fe(2) sites and Fe(2) and Fe(3) become much larger than the others. The first principle DFT calculations also support the spin model expected from the bond angle^25^. The dominant exchange indicated by the DFT calculations is shown as thick solid ($J_\mathrm {bb}$ = 97.4 K), dashed ($J_\mathrm {vb2}$ = 88.3 K), and dotted ($J_\mathrm {vb1}$ = 42.2 K), thin solid ($J_\mathrm {vv}$ = 14.0 K) lines in Fig. 5. (The schematic view of the exchange parameters and the antiferromagnetic structure is also illustrated in Fig. S4.) The antiferromagnetic structure should be stabilized by strongly antiferromagnetic $J_\mathrm {bb}$ at an Fe(1)–Fe(2) bond and $J_\mathrm {vb2}$ at an Fe(2)–Fe(3) bond, which is two times as large as $J_\mathrm {vb1}$ at an Fe(1)–Fe(3) bond. The dominant antiferromagnetic exchange couplings give rise to a sawtooth antiferromagnetic chain along the *a*-direction.

The canting angle between magnetic moments is much smaller than those in the other sawtooth chain magnets^18,22^. This suggests that a single-ion anisotropy is small and antiferromagnetic exchange couplings favor the magnetic structure with the small noncollinearity. The magnetic moment at the Fe(1) and Fe(2) sites is canted from the *b*-direction, and also contains the *a*-component. Due to the twofold rotation axis along the *c*-axis at the Fe(1) and Fe(2) sites, the *c*-component of the *D*-vector, characterizing the Dzyaloshinskii-Moriya interactions, is required to be staggered along the chain. The staggered Dzyaloshinskii-Moriya interactions may induce the noncollinearity of the antiferromagnetic structure.

**Figure 8:**
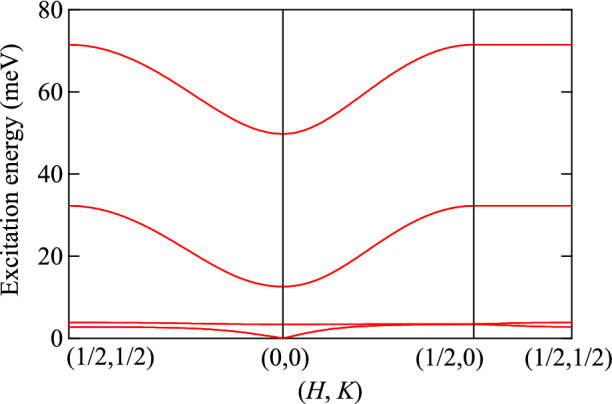
Dispersion relation derived from the collinear antiferromagnetic structure and the exchange parameters of $J_\mathrm {vb1}$ = 42.2 K (Fe(1)–Fe(3)), $J_\mathrm {vv}$ = 14.0 K (Fe(3)–Fe(3)), $J_\mathrm {vb2}$ = 88.3 K (Fe(2)–Fe(3)), and $J_\mathrm {bb} =~97.4$ K (Fe(1)–Fe(2)).

The possible dispersion relations of the antiferromagnetic magnons are estimated by the linear spin-wave approximation. Figure 8 shows the expected dispersion relation based on the exchange parameters estimated from DFT calculations. The antiferromagnetic structure used for the calculation is simplified to be a collinear antiferromagnetic structure, as shown in Fig. S4. The excitation spectrum consists of sixteen magnon branches due to the same number of magnetic atoms included in the magnetic unit cell. The branch is fourfold degenerate; the degeneracy is induced from the presence of the two decoupled layers along the *c*-axis, and the space-time inversion symmetry that is present at the center of Fe(3)–Fe(3) bonds. As a result, four modes appear at 0-3.4, 3.4–3.8, 12.6–32.2, and 49.8–71.5 meV. One of the branch becomes nearly flat at the excitation energy of 3.4–3.8 meV.

The above calculation indicates that sawtooth chain antiferromagnet can possess magnons with nearly flat dispersion relation. In the Raman scattering study, some peaks were observed in the chain specific polarization at 6.8, 20.7, 64.8, and 76.9 meV^24^. The peaks have been interpreted as two magnon scattering from the nearly flat bands along the chain direction. The sharp one at 6.8 meV may correspond to the two magnon scattering of the nearly flat modes at 3.4–3.8 meV. On the other hand, the above spin wave calculation cannot explain the others, such as the sharp mode observed at 64.8 meV. The estimate of the exchange parameters may not be accurate enough to reproduce the flat magnon bands at high energies. To assign these excitations, precise determination of the magnetic exchange parameters would be necessary.

## Conclusion

The magnetic structure of the sawtooth-chain antiferromagnet $\hbox {Fe}_2\hbox {Se}_2\hbox {O}_7$ was found from the single crystalline and powder neutron diffraction experiments. The nearly collinear antiferromagnetic structure with the magnetic moments aligned along the *b*-axis should be stabilized by the dominant antiferromagnetic exchange between Fe(1)–Fe(2) and Fe(2)–Fe(3) sites. The possible flat dispersion relation of low-energy magnons is suggested by the linear spin wave calculation.

## Methods

We performed magnetization measurements and neutron diffraction experiments to reveal the magnetic structure. Single crystalline samples were grown by the chemical vapor transport method^24^, while polycrystalline samples were prepared by the hydrothermal synthesis^25^. The magnetization measurement was performed by using VSM option of Physical Properties Measurement System (Quantum Design). The magnetization was measured in the temperature range 2–300 K and the external magnetic field up to 9 T. The measurement was performed with increasing the temperature after a field-cooling protocol with a magnetic field of 0.1 T. A single crystalline sample with the approximate size 3 $\times$ 1 $\times$ 0.5 mm was mounted on the quartz sample holder so that the crystallographic *a*, *b* or *c*-axis become parallel to a magnetic field direction. The orientation of the single crystalline sample was determined using the X-ray diffractometer (Rigaku Ultima IV diffractometer) with Cu $K_\alpha$ radiation in the geometry of a parallel beam with a thin-film attachment.

The magnetic structure was investigated through single crystalline and powder neutron diffraction experiments. A single crystal with the size of 0.5 $\times$ 0.5 $\times$ 1 $\hbox {mm}^3$ was used for the single crystal diffraction experiments using KOALA white-beam neutron Laue diffractometer^32^. Image data with the different crystal orientation were collected at several temperatures between 4–120 K, and the room temperature. The temperature was controlled by a bottom-loading cryostat between 4 and 120 K. Indexing, intensity integration, and wavelength distribution normalization were performed using the LAUEG software^33^. A crystal and magnetic structure refinement was carried out using JANA2006 software suite^34^.

The powder neutron diffraction experiments were performed using high-resolution powder diffractometer ECHIDNA^35^. 2.335 g of the powder sample was loaded into a vanadium can with a diameter of 9 mm. To confirm the crystal structure, neutrons with the wavelength of 1.6220 Å were selected by the monochromator using Ge 335 reflections. The 10’ secondary collimator was used for better resolution. To collect magnetic reflections, neutrons with the wavelength of 2.4395 Å were selected by the monochromator using Ge 331 reflection. No secondary collimator was used to increase statistics. The temperature was controlled by a top-loading cryostat between 3 and 120 K. Rietveld analyses were performed by the Fullprof software suite^36^. The two powder diffraction patterns collected with the two wavelengths are simultaneously refined for 120 K.

$^{57}$Fe Mössbauer spectra were further analyzed to complement the neutron diffraction experiments on $\hbox {Fe}_2\hbox {Se}_2\hbox {O}_7$. The $^{57}$Fe Mössbauer experiments were performed in transmission geometry with a commercial MS1104EM setup using an 1850 MBq $\gamma$-source of $^{57}$Co(Rh) equipped with Janis Research SHI-850-1 cryostat operating in the range 12–325 K^25^. The electric field gradient (EFG) tensor was calculated based on the crystal structure determined from the KOALA experiments at 4 K. Then, the direction of the hyperfine fields against the electric field gradient (EFG) tensor was estimated by using the previous experimental data^25^. The spectra were fitted using the SpectrRelax program^37^. The isomer shift values are referred to that of $\alpha$-Fe at room temperature.

## Supplementary information

Supplementary Information.
