# Supplementary material for: Magnetic structure study of the sawtooth chain antiferromagnet Fe2Se2O7
Source: Sci Rep. 2021 Dec 15;11:24049. doi: 10.1038/s41598-021-03058-5 (PMC8674342; doi:10.1038/s41598-021-03058-5)
Supplement: Supplementary file 1 — Supplementary Information. [file 41598_2021_3058_MOESM1_ESM.pdf]

Supplementary information of Magnetic structure study of the sawtooth chain antiferromagnet $Fe_2Se_2O_7$

Kazuhiro Nawa[1,*], Maxim Avdeev[2,3], Peter Berdonosov[4], Alexey Sobolev[4], Igor Presniakov[4], Alena Aslandukova[4,5], Ekaterina Kozlyakova[4,6,+], Alexander Vasiliev[4,6], Igor Shchetinin[6], and Taku J Sato[1]

[1]Institute of Multidisciplinary Research for Advanced Materials, Tohoku University, 2-1-1 Katahira, Sendai 980-8577, Japan
[2]Australian Centre for Neutron Research, Australian Nuclear Science and Technology Organisation, Kirrawee DC, NSW 2232, Australia
[3]School of Chemistry, The University of Sydney, Sydney 2006, Australia
[4]Lomonosov Moscow State University, Moscow 119991, Russia
[5]University of Bayreuth, Bavarian Research Institute of Experimental Geochemistry and Geophysics, Bayreuth 95447, Germany
[6]National University of Science and Technology MISIS, 119991 Moscow, Russia
[*]knawa@tohoku.ac.jp
[+]evenuel1@gmail.com

Supplementary figures of the powder and single crystalline neutron diffraction experiments

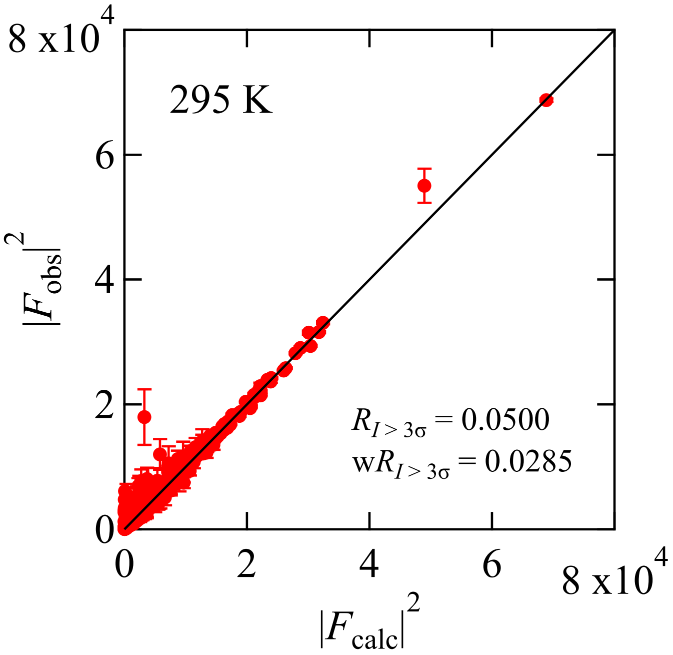

Figure S1. $|F_{obs}|^2$ vs $|F_{calc}|^2$ plot for (a) 4 K (b) 60 K (c) 90 K, (d) 120 K and (e) 295 K. The refinement parameters are listed in Table S1.

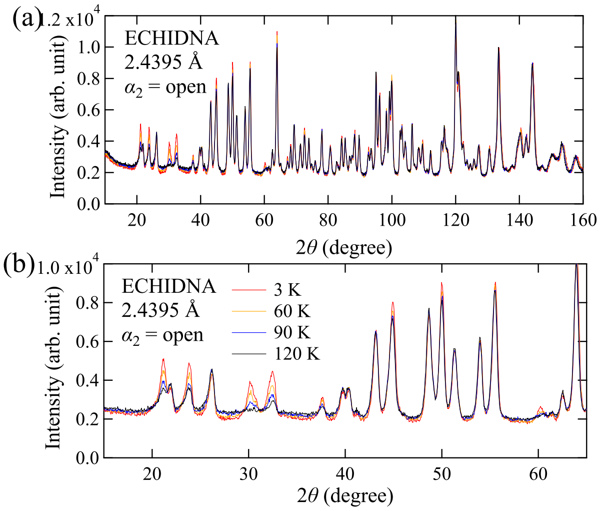

Figure S2. (a) Powder neutron diffraction patterns measured at 3, 60, 90 and 120 K. (b) Enlarged view at low angles.

Refinement parameters, structural parameters and list of basis vectors

All the refinement and structural parameters found in the powder and single crystalline neutron diffraction experiments are listed in this section. Table S1 shows the refinement parameters and Tables S2, S3, S4, S5, and S6 represent the structural parameters estimated from the single crystalline neutron diffraction experiments performed at 4, 60, 90, 120 and 295 K, respectively. Table S7 shows the refinement parameters and Tables S8, S9, S10, and S11 represent those estimated from the powder neutron diffraction experiments performed at 3, 60, 90, and 120 K, respectively. In addition, a list of basis vectors, which is used for the magnetic structure refinement, is shown in Table S13.

Table S1. Crystallographic data and refinement parameters of the single crystal diffraction experiments at different temperatures.

	4 K	60 K	90 K	120 K	295 K
a (Å)	6.64	6.64	6.64	6.64	6.64
b (Å)	12.85	12.85	12.85	12.84	12.83
c (Å)	13.25	13.26	13.27	13.27	13.28
V (Å^3)	1131.2	1131.2	1131.3	1131.3	1131.3
Z			8		
$C_{\mathrm{Fe1},a}$ (μ_B)	−0.94(12)	−0.82(12)	−0.74(16)	–	–
$C_{\mathrm{Fe1},b}$ (μ_B)	4.47(10)	3.80(11)	3.17(14)	–	–
$C_{\mathrm{Fe2},a}$ (μ_B)	1.60(11)	1.44(13)	1.38(15)	–	–
$C_{\mathrm{Fe2},b}$ (μ_B)	3.96(10)	3.41(11)	2.88(15)	–	–
$C_{\mathrm{Fe3},a}$ (μ_B)	−0.22(6)	−0.25(5)	−0.32(9)	–	–
$C_{\mathrm{Fe3},b}$ (μ_B)	−4.37(8)	−3.29(8)	−2.52(11)	–	–
$C_{\mathrm{Fe3},c}$ (μ_B)	0.6(2)	0.87(19)	0.7(3)	–	–
Calculated density (g/cm^3)	4.481	4.481	4.480	4.480	4.480
minimum h range	−14	−14	−14	−14	−14
maximum h range	14	14	14	14	14
minimum k range	−14	−14	−14	−14	−27
maximum k range	14	14	14	14	17
minimum l range	−28	−28	−28	−28	−16
maximum l range	23	23	20	8	19
$F(000)$			603.688		
Reflections collected	33951	33993	31324	23344	33770
Independent reflections	2704	2685	2652	2309	2847
Dataootnotemark[1]/Restraints/parameters	1528/0/54	1489/0/54	1433/0/54	1287/0/46	1488/0/102
R indices (R_w[b])	0.0719	0.0792	0.0879	0.0796	0.0496
R indices (wR_w[b])	0.0522	0.0528	0.0532	0.0434	0.0285
Goodness of fit on F	2.30	2.29	2.27	1.76	1.33

[a] Reflections with $I > 3\sigma$ and $\sin\theta/\lambda < 1$ are used for the refinement.
[b] $R = \sum||F_{\mathrm{obs}}| - |F_{\mathrm{calc}}||/\sum|F_{\mathrm{obs}}|$, $wR = \{\sum w(|F_{\mathrm{obs}}| - |F_{\mathrm{calc}}|)^2/\sum w|F_{\mathrm{obs}}|^2\}^{1/2}$, $w = 1/(\sigma^2|F_{\mathrm{obs}}| + 0.0001|F_{\mathrm{obs}}^2|)$.

Table S2. Structure parameters of $Fe_2Se_2O_7$ at 4 K determined from the single crystal diffraction experiment. The lattice parameter is a = 6.64 Å, b = 12.85 Å, c = 13.25 Å. The atomic coordinations are represented by fractional coordinates. The equivalent isotropic displacement parameters U_{iso} are listed in a unit of Å^2. Occupancy is fixed to 1 for all atoms.

atom	site	x	y	z	100 U_{iso}
Fe(1)	4c	0.25	0.25	0.33617(10)	0.10(2)
Fe(2)	4d	0.25	0.75	0.21721(10)	0.17(3)
Fe(3)	8e	0.03556(14)	0.62435(11)	0.02337(7)	0.141(17)
Se(1)	8e	0.01148(15)	0.45269(15)	0.22129(8)	0.17(2)
Se(2)	8e	0.02972(17)	0.13230(14)	0.04345(8)	0.170(19)
O(1)	8e	0.0281(2)	0.7078(2)	0.14450(11)	0.27(3)
O(2)	8e	0.1956(2)	0.3630(2)	0.23474(12)	0.41(3)
O(3)	8e	0.7954(2)	0.3815(2)	0.22037(11)	0.40(3)
O(4)	8e	0.0234(3)	0.4777(2)	0.09289(12)	0.38(3)
O(5)	8e	−0.0195(2)	0.2587(2)	0.07015(13)	0.46(3)
O(6)	8e	0.8317(2)	0.09968(20)	−0.03184(11)	0.39(3)
O(7)	8e	0.2176(2)	0.13876(19)	−0.04763(11)	0.33(3)

Table S3. Structure parameters of $Fe_2Se_2O_7$ at 60 K determined from the single crystal diffraction experiment. The lattice parameter is a = 6.64 Å, b = 12.85 Å, c = 13.26 Å. The atomic coordinations are represented by fractional coordinates. The equivalent isotropic displacement parameters U_{iso} are listed in a unit of Å^2. Occupancy is fixed to 1 for all atoms.

atom	site	x	y	z	100 U_{iso}
Fe(1)	4c	0.25	0.25	0.33602(10)	0.20(3)
Fe(2)	4d	0.25	0.75	0.21687(10)	0.22(3)
Fe(3)	8e	0.03565(15)	0.62426(12)	0.02345(7)	0.208(18)
Se(1)	8e	0.01145(16)	0.45253(16)	0.22124(8)	0.23(2)
Se(2)	8e	0.02953(17)	0.13234(14)	0.04333(8)	0.22(2)
O(1)	8e	0.0278(2)	0.7076(2)	0.14450(11)	0.36(3)
O(2)	8e	0.1957(3)	0.3629(2)	0.23472(12)	0.46(3)
O(3)	8e	0.7954(2)	0.3813(2)	0.22042(12)	0.47(3)
O(4)	8e	0.0234(3)	0.4776(2)	0.09284(13)	0.43(3)
O(5)	8e	−0.0191(2)	0.2589(2)	0.07002(14)	0.52(3)
O(6)	8e	0.8316(2)	0.0997(2)	−0.03199(12)	0.47(3)
O(7)	8e	0.2176(2)	0.1386(2)	−0.04770(12)	0.37(3)

Table S4. Structure parameters of $Fe_2Se_2O_7$ at 90 K determined from the single crystal diffraction experiment. The lattice parameter is a = 6.64 Å, b = 12.85 Å, c = 13.27 Å. The atomic coordinations are represented by fractional coordinates. The equivalent isotropic displacement parameters U_{iso} are listed in a unit of Å^2. Occupancy is fixed to 1 for all atoms.

atom	site	x	y	z	100 U_{iso}
Fe(1)	4c	0.25	0.25	0.33588(11)	0.25(3)
Fe(2)	4d	0.25	0.75	0.21658(11)	0.28(3)
Fe(3)	8e	0.03553(15)	0.62427(12)	0.02350(7)	0.261(19)
Se(1)	8e	0.01135(17)	0.45256(16)	0.22116(9)	0.27(2)
Se(2)	8e	0.02941(18)	0.13250(15)	0.04308(9)	0.28(2)
O(1)	8e	0.0275(2)	0.7076(2)	0.14451(12)	0.38(3)
O(2)	8e	0.1957(3)	0.3628(2)	0.23462(13)	0.52(3)
O(3)	8e	0.7955(3)	0.3811(2)	0.22044(12)	0.53(3)
O(4)	8e	0.0232(3)	0.4775(2)	0.09293(13)	0.43(3)
O(5)	8e	−0.0190(3)	0.2587(2)	0.06985(14)	0.56(3)
O(6)	8e	0.8315(2)	0.1001(2)	−0.03214(12)	0.53(3)
O(7)	8e	0.2175(2)	0.1385(2)	−0.04787(12)	0.41(3)

Table S5. Structure parameters of $Fe_2Se_2O_7$ at 120 K determined from the single crystal diffraction experiment. The lattice parameter is a = 6.64 Å, b = 12.84 Å, c = 13.27 Å. The atomic coordinations are represented by fractional coordinates. The equivalent isotropic displacement parameters U_{iso} are listed in a unit of Å^2. Occupancy is fixed to 1 for all atoms.

atom	site	x	y	z	100 U_{iso}
Fe(1)	4c	0.25	0.25	0.33569(9)	0.31(2)
Fe(2)	4d	0.25	0.75	0.21620(9)	0.39(3)
Fe(3)	8e	0.03551(14)	0.62410(11)	0.02360(6)	0.327(16)
Se(1)	8e	0.01131(15)	0.45233(14)	0.22108(7)	0.36(2)
Se(2)	8e	0.02908(16)	0.13254(13)	0.04281(7)	0.349(18)
O(1)	8e	0.0271(2)	0.70728(17)	0.14469(10)	0.44(3)
O(2)	8e	0.1954(2)	0.36283(19)	0.23446(11)	0.69(3)
O(3)	8e	0.7955(2)	0.38061(18)	0.22042(10)	0.66(3)
O(4)	8e	0.0230(2)	0.47749(19)	0.09281(11)	0.52(3)
O(5)	8e	−0.0190(2)	0.25881(18)	0.06973(12)	0.66(3)
O(6)	8e	0.8313(2)	0.10025(18)	−0.03244(10)	0.66(3)
O(7)	8e	0.2170(2)	0.13847(18)	−0.04789(10)	0.51(3)

Table S6. Structure parameters of $Fe_2Se_2O_7$ at 295 K determined from the single crystal diffraction experiment. The lattice parameter is a = 6.64 Å, b = 12.83 Å, c = 13.28 Å. The atomic coordinations are represented by fractional coordinates. The anisotropic displacement parameters U_{ij} are listed in a unit of Å^2. Occupancy is fixed to 1 for all atoms.

atom	site	x	y	z	100 U_{eq}
Fe(1)	4c	0.25	0.25	0.33454(8)	0.76(3)
Fe(2)	4d	0.25	0.75	0.21486(9)	0.87(3)
Fe(3)	8e	0.03439(9)	0.62383(5)	0.02426(6)	0.768(16)
Se(1)	8e	0.01091(9)	0.45150(5)	0.22038(8)	0.83(2)
Se(2)	8e	0.02785(9)	0.13287(5)	0.04122(7)	0.78(2)
O(1)	8e	0.02504(12)	0.70677(7)	0.14536(9)	0.91(3)
O(2)	8e	0.19484(16)	0.36280(9)	0.23378(10)	1.37(3)
O(3)	8e	0.79650(14)	0.37960(8)	0.22024(11)	1.31(3)
O(4)	8e	0.02137(13)	0.47700(7)	0.09261(10)	0.92(3)
O(5)	8e	−0.01818(13)	0.25900(7)	0.06823(11)	1.17(3)
O(6)	8e	0.83010(12)	0.10187(7)	−0.03393(11)	1.40(3)
O(7)	8e	0.21551(12)	0.13805(7)	−0.04891(10)	0.98(3)

atom	site	100 U_{11}	100 U_{22}	100 U_{33}	100 U_{12}	100 U_{23}	100 U_{31}
Fe(1)	4c	0.55(3)	0.90(3)	0.83(6)	−0.073(20)	0	0
Fe(2)	4d	0.55(3)	0.80(3)	1.26(6)	−0.07(2)	0	0
Fe(3)	8e	0.826(17)	0.60(2)	0.88(4)	0.005(18)	−0.01(2)	−0.04(2)
Se(1)	8e	0.90(2)	0.88(3)	0.73(5)	−0.058(18)	−0.01(2)	0.05(3)
Se(2)	8e	0.75(2)	0.67(3)	0.92(6)	−0.025(19)	−0.01(3)	0.08(3)
O(1)	8e	0.68(3)	1.03(4)	1.01(8)	0.10(3)	−0.08(3)	−0.19(4)
O(2)	8e	1.06(4)	1.59(5)	1.45(8)	0.20(3)	−0.11(4)	0.53(4)
O(3)	8e	0.88(3)	1.43(4)	1.61(7)	−0.18(3)	0.13(4)	0.53(5)
O(4)	8e	1.20(3)	0.74(4)	0.82(8)	−0.12(3)	0.00(4)	0.11(4)
O(5)	8e	1.52(4)	0.82(4)	1.15(8)	0.04(3)	0.06(4)	−0.06(4)
O(6)	8e	0.71(3)	1.61(4)	1.87(8)	−0.18(3)	0.00(4)	−0.51(4)
O(7)	8e	0.73(3)	1.02(4)	1.20(7)	0.06(3)	0.08(3)	0.10(4)

Table S7. Crystallographic data and refinement parameters of the powder neutron diffraction experiments at different temperatures.

temperature (K)	3	60	90	120	
wavelength (Å)	2.4395	2.4395	2.4395	2.4395	1.6220
a (Å)	6.6154(1)	6.6168(2)	6.6191(2)	6.6202(4)	
b (Å)	12.8137(2)	12.8143(2)	12.8166(3)	12.8176(7)	
c (Å)	13.2522(3)	13.2573(6)	13.2655(7)	13.2698(11)	
V (Å^3)	1123.36(4)	1124.08(6)	1125.36(7)	1126.01(13)	
$C_{\mathrm{Fe(1)},a}$ (μ_B)	−0.90(2)	−0.69(2)	−0.34(4)	–	
$C_{\mathrm{Fe(1)},b}$ (μ_B)	4.00(7)	2.93(9)	1.56(17)	–	
$C_{\mathrm{Fe(2)},a}$ (μ_B)	1.52(4)	1.47(5)	1.01(9)	–	
$C_{\mathrm{Fe(2)},b}$ (μ_B)	3.49(8)	3.17(10)	2.87(16)	–	
$C_{\mathrm{Fe(3)},a}$ (μ_B)	−0.177(3)	−0.176(7)	−0.12(2)	–	
$C_{\mathrm{Fe(3)},b}$ (μ_B)	−3.61(7)	−2.33(9)	−0.93(16)	–	
$C_{\mathrm{Fe(3)},c}$ (μ_B)	0.509(10)	0.62(2)	0.28(5)	–	
Calculated density (g/cm^3)	4.51	4.51	4.50	4.50	
No. of parameters	70[a]	70[a]	70[a]	105	
2θ range used for refinement (°)	6.0–163.9	6.0–163.9	6.0–163.9	6.0–163.9	6.0–158.0
No. of nuclear reflections	315	315	317	315	1059
No. of magnetic reflections	1244	1244	1252	–	–
R_p (%)	7.65	7.84	8.20	8.67	9.31
R_wp (%)	8.67	8.76	9.01	9.27	10.40
R_e (%)	4.20	4.35	4.52	4.64	5.43
χ^2	4.25	4.06	3.98	3.99	3.68
R_mag	3.54	4.66	4.87	–	–

[a] Shift parameters are not included since they are not refined in the final refinement.

Table S8. Structure parameters of $Fe_2Se_2O_7$ at 3 K determined from the powder diffraction experiment. The space group is *Pccn*, and the lattice parameter is a = 6.6154(1) Å, b = 12.8137(2) Å, c = 13.2522(3) Å. The atomic coordinations are represented by fractional coordinates. The equivalent isotropic displacement parameters B_{iso} are listed in a unit of Å^2. Occupancy is fixed to 1 for all atoms.

atom	site	x	y	z	B_{iso}
Fe(1)	4c	0.25	0.25	0.3341(4)	0.17(6)
Fe(2)	4d	0.25	0.75	0.2163(5)	0.17(6)
Fe(3)	8e	0.0363(4)	0.6232(3)	0.0238(2)	0.17(6)
Se(1)	8e	0.0096(8)	0.4511(3)	0.2212(3)	0.55(7)
Se(2)	8e	0.0251(7)	0.1326(4)	0.0408(3)	0.55(7)
O(1)	8e	0.0248(9)	0.7088(4)	0.1451(5)	0.28(6)
O(2)	8e	0.1948(8)	0.3643(5)	0.2348(5)	0.28(6)
O(3)	8e	0.7979(10)	0.3801(5)	0.2203(4)	0.28(6)
O(4)	8e	0.0232(10)	0.4772(4)	0.0912(4)	0.28(6)
O(5)	8e	−0.0146(10)	0.2584(4)	0.0697(4)	0.28(6)
O(6)	8e	0.8296(6)	0.1005(4)	−0.0311(5)	0.28(6)
O(7)	8e	0.2181(8)	0.1380(5)	−0.0489(5)	0.28(6)

Table S9. Structure parameters of $Fe_2Se_2O_7$ at 60 K determined from the powder diffraction experiment. The space group is *Pccn*, and the lattice parameter is a = 6.6168(2) Å, b = 12.8143(2) Å, c = 13.2573(6) Å. The atomic coordinations are represented by fractional coordinates. The equivalent isotropic displacement parameters B_{iso} are listed in a unit of Å^2. Occupancy is fixed to 1 for all atoms.

atom	site	x	y	z	B_{iso}
Fe(1)	4c	0.25	0.25	0.3339(4)	0.24(6)
Fe(2)	4d	0.25	0.75	0.2150(5)	0.24(6)
Fe(3)	8e	0.0363(4)	0.6234(3)	0.0234(2)	0.24(6)
Se(1)	8e	0.0104(8)	0.4509(3)	0.2215(3)	0.66(7)
Se(2)	8e	0.0253(7)	0.1314(4)	0.0404(3)	0.66(7)
O(1)	8e	0.0232(9)	0.7088(4)	0.1450(4)	0.37(5)
O(2)	8e	0.1950(8)	0.3642(4)	0.2337(5)	0.37(5)
O(3)	8e	0.7979(10)	0.3802(5)	0.2209(4)	0.37(5)
O(4)	8e	0.0227(10)	0.4775(4)	0.0917(4)	0.37(5)
O(5)	8e	−0.0145(10)	0.2586(4)	0.0697(4)	0.37(5)
O(6)	8e	0.8295(6)	0.1005(4)	−0.0305(5)	0.37(5)
O(7)	8e	0.2180(8)	0.1382(5)	−0.0491(5)	0.37(5)

Table S10. Structure parameters of $Fe_2Se_2O_7$ at 90 K determined from the powder diffraction experiment. The space group is *Pccn*, and the lattice parameter is a = 6.6191(2) Å, b = 12.8166(3) Å, c = 13.2655(7) Å. The atomic coordinations are represented by fractional coordinates. The equivalent isotropic displacement parameters B_{iso} are listed in a unit of Å^2. Occupancy is fixed to 1 for all atoms.

atom	site	x	y	z	B_{iso}
Fe(1)	4c	0.25	0.25	0.3337(4)	0.43(6)
Fe(2)	4d	0.25	0.75	0.2148(5)	0.43(6)
Fe(3)	8e	0.0353(4)	0.6227(3)	0.0238(2)	0.43(6)
Se(1)	8e	0.0094(8)	0.4509(3)	0.2206(3)	0.78(7)
Se(2)	8e	0.0250(7)	0.1318(4)	0.0413(3)	0.78(7)
O(1)	8e	0.0254(9)	0.7089(4)	0.1442(4)	0.62(5)
O(2)	8e	0.1949(8)	0.3644(4)	0.2338(5)	0.62(5)
O(3)	8e	0.7977(9)	0.3810(5)	0.2212(4)	0.62(5)
O(4)	8e	0.0220(10)	0.4763(4)	0.0921(4)	0.62(5)
O(5)	8e	−0.0150(10)	0.2597(4)	0.0702(4)	0.62(5)
O(6)	8e	0.8291(6)	0.1014(4)	−0.0295(5)	0.62(5)
O(7)	8e	0.2184(8)	0.1379(5)	−0.0491(5)	0.62(5)

Table S11. Structure parameters of $Fe_2Se_2O_7$ at 120 K determined from the powder diffraction experiment. The space group is *Pccn*, and the lattice parameter is a = 6.6202(4) Å, b = 12.8176(7) Å, c = 13.2698(11) Å. The atomic coordinations are represented by fractional coordinates. The equivalent isotropic displacement parameters B_{iso} are listed in a unit of Å^2. Occupancy is fixed to 1 for all atoms.

atom	site	x	y	z	B_{iso}
Fe(1)	4c	0.25	0.25	0.3333(4)	0.21
Fe(2)	4d	0.25	0.75	0.2136(4)	0.29
Fe(3)	8e	0.0343(4)	0.6239(3)	0.0243(2)	0.24
Se(1)	8e	0.0120(7)	0.4519(3)	0.2212(3)	0.31(7)
Se(2)	8e	0.0260(7)	0.1317(4)	0.0420(3)	0.64(9)
Se(2)	8e	0.0263(9)	0.7082(4)	0.1458(4)	0.64(12)
O(2)	8e	0.1966(10)	0.3643(5)	0.2363(5)	0.84(13)
O(3)	8e	0.7973(9)	0.3804(5)	0.2206(4)	0.19(11)
O(4)	8e	0.0223(10)	0.4778(4)	0.0933(4)	0.32(10)
O(5)	8e	−0.0151(10)	0.2604(4)	0.0684(4)	0.23(12)
O(6)	8e	0.8314(7)	0.1023(4)	−0.0312(5)	0.37(12)
O(7)	8e	0.2189(8)	0.1382(5)	−0.0476(5)	0.50(11)

Table S12. List of the Fe sites in a primitive unit cell.

Fe(1)-1	x	y	z
Fe(1)-2	$-x+1$	$y+1/2$	$-z+1/2$
Fe(1)-3	$-x+1$	$-y+1$	$-z+1$
Fe(1)-4	x	$-y+1/2$	$z+1/2$
Fe(2)-1	x	y	z
Fe(2)-2	$-x+1$	$y-1/2$	$-z+1/2$
Fe(2)-3	$-x+1$	$-y+1$	$-z+1$
Fe(2)-4	x	$-y+3/2$	$z+1/2$
Fe(3)-1	x	y	z
Fe(3)-2	$-x+1/2$	$-y+3/2$	z
Fe(3)-3	$-x+1$	$y-1/2$	$-z+1/2$
Fe(3)-4	$x+1/2$	$-y+1$	$-z+1/2$
Fe(3)-5	$-x+1$	$-y+1$	$-z+1$
Fe(3)-6	$x+1/2$	$y-1/2$	$-z+1$
Fe(3)-7	x	$-y+3/2$	$z+1/2$
Fe(3)-8	$-x+1/2$	y	$z+1/2$

Table S13. List of basis vectors of all the irreducible representations (IRs, Γ_1–Γ_8) for the three inequivalent Fe sites in $Fe_2Se_2O_7$ with the space group *Pccn* and the magnetic modulation vector of $\mathbf{k}$ = (0,0,0). All the IRs are one dimensional. The Fe(1)-1, Fe(2)-1, Fe(3)-1 sites are defined to be the atomic site listed in Table. S2–S4 and S8–S10, such as (0.25, 0.25, 0.33617), (0.25, 0.75, 0.21721), and (0.03556, 0.62435, 0.02337) at 4 K, respectively. The rest of the Fe sites follow the definition given in Table. S12.

IRs	Fe(1)-1/Fe(2)-1	Fe(1)-2/Fe(2)-2	Fe(1)-3/Fe(2)-3	Fe(1)-4/Fe(2)-4
Γ_1	0 0 1	0 0 −1	0 0 1	0 0 −1
Γ_2	0 0 1	0 0 −1	0 0 −1	0 0 1
Γ_3	0 0 1	0 0 1	0 0 1	0 0 1
Γ_4	0 0 1	0 0 1	0 0 −1	0 0 −1
Γ_5	1 0 0	−1 0 0	1 0 0	−1 0 0
	0 1 0	0 1 0	0 1 0	0 1 0
Γ_6	1 0 0	−1 0 0	−1 0 0	1 0 0
	0 1 0	0 1 0	0 −1 0	0 −1 0
Γ_7	1 0 0	1 0 0	1 0 0	1 0 0
	0 1 0	0 −1 0	0 1 0	0 −1 0
Γ_8	1 0 0	1 0 0	−1 0 0	−1 0 0
	0 1 0	0 −1 0	0 −1 0	0 1 0

IRs	Fe(3)-1	Fe(3)-2	Fe(3)-3	Fe(3)-4	Fe(3)-5	Fe(3)-6	Fe(3)-7	Fe(3)-8
Γ_1	1 0 0	−1 0 0	−1 0 0	1 0 0	1 0 0	−1 0 0	−1 0 0	1 0 0
	0 1 0	0 −1 0	0 1 0	0 −1 0	0 1 0	0 −1 0	0 1 0	0 −1 0
	0 0 1	0 0 1	0 0 −1	0 0 −1	0 0 1	0 0 1	0 0 −1	0 0 −1
Γ_2	1 0 0	−1 0 0	−1 0 0	1 0 0	−1 0 0	1 0 0	1 0 0	−1 0 0
	0 1 0	0 −1 0	0 1 0	0 −1 0	0 −1 0	0 1 0	0 −1 0	0 1 0
	0 0 1	0 0 1	0 0 −1	0 0 −1	0 0 −1	0 0 −1	0 0 1	0 0 1
Γ_3	1 0 0	−1 0 0	1 0 0	−1 0 0	1 0 0	−1 0 0	1 0 0	−1 0 0
	0 1 0	0 −1 0	0 −1 0	0 1 0	0 1 0	0 −1 0	0 −1 0	0 1 0
	0 0 1	0 0 1	0 0 1	0 0 1	0 0 1	0 0 1	0 0 1	0 0 1
Γ_4	1 0 0	−1 0 0	1 0 0	−1 0 0	−1 0 0	1 0 0	−1 0 0	1 0 0
	0 1 0	0 −1 0	0 −1 0	0 1 0	0 −1 0	0 1 0	0 1 0	0 −1 0
	0 0 1	0 0 1	0 0 1	0 0 1	0 0 −1	0 0 −1	0 0 −1	0 0 −1
Γ_5	1 0 0	1 0 0	−1 0 0	−1 0 0	1 0 0	1 0 0	−1 0 0	−1 0 0
	0 1 0	0 1 0	0 1 0	0 1 0	0 1 0	0 1 0	0 1 0	0 1 0
	0 0 1	0 0 −1	0 0 −1	0 0 1	0 0 1	0 0 −1	0 0 −1	0 0 1
Γ_6	1 0 0	1 0 0	−1 0 0	−1 0 0	−1 0 0	−1 0 0	1 0 0	1 0 0
	0 1 0	0 1 0	0 1 0	0 1 0	0 −1 0	0 −1 0	0 −1 0	0 −1 0
	0 0 1	0 0 −1	0 0 −1	0 0 1	0 0 −1	0 0 1	0 0 1	0 0 −1
Γ_7	1 0 0	1 0 0	1 0 0	1 0 0	1 0 0	1 0 0	1 0 0	1 0 0
	0 1 0	0 1 0	0 −1 0	0 −1 0	0 1 0	0 1 0	0 −1 0	0 −1 0
	0 0 1	0 0 −1	0 0 1	0 0 −1	0 0 1	0 0 −1	0 0 1	0 0 −1
Γ_8	1 0 0	1 0 0	1 0 0	1 0 0	−1 0 0	−1 0 0	−1 0 0	−1 0 0
	0 1 0	0 1 0	0 −1 0	0 −1 0	0 −1 0	0 −1 0	0 1 0	0 1 0
	0 0 1	0 0 −1	0 0 1	0 0 −1	0 0 −1	0 0 1	0 0 −1	0 0 1

Supplementary table and figure of the Mössbauer spectroscopy

Table S14. Values of the principal components V_{ii}^{tot} of the EFG tensor, the asymmetry parameters η^{theor}, and the theoretical quadrupole splittings Δ^{theor} calculated from the crystal structure determined at 4 K.

Site	V_{XX}^{tot}	V_{YY}^{tot}	V_{ZZ}^{tot}	η^{theor}	Δ^{theor}
Fe(1)	0.32837	0.43619	−0.76455	0.141	1.214
Fe(2)	0.07405	0.67315	−0.74721	0.802	1.303
Fe(3)	−0.31389	−0.04420	0.35808	−0.753	0.618

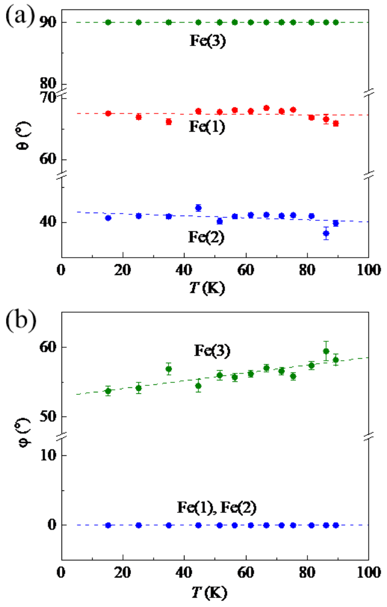

Figure S3. Temperature dependences of polar angles (θ, ψ), which specify the direction of hyperfine field B_{hf} with respect to the principal axes of EFG.

Linear spin wave approximation on the collinear antiferromagnetic structure

In this section, we discuss the dispersion relation expected for the antiferromagnetic structure in $Fe_2Se_2O_7$. The dominant exchange couplings are J_{vb1} = 42.2 K (Fe(1)–Fe(3)), J_{vv} = 14.0 K (Fe(3)–Fe(3)), J_{vb2} = 88.3 K (Fe(2)–Fe(3)), and J_{bb} = 97.4 K (Fe(1)–Fe(2)) according to the DFT calculations[1]. Interlayer couplings between neighboring *ab*-planes and exchange couplings connecting next-nearest neighbors are ignored. The model Hamiltonian can be constructed from the 8 nonequivalent sublattices in the magnetic unit cell, as illustrated in Figure 4. The Hamiltonian is given by

$$H = \frac{1}{2}\sum_{A(\neq B)}\sum_{B}(H_{\mathrm{AB}} + H_{\mathrm{BA}}), \tag{1}$$

$$H_{\mathrm{AB}} = \sum_{i\in A}\sum_{j\in B} J_{ij}\mathbf{S}_i\cdot\mathbf{S}_j, \tag{2}$$

where A and B represent one of the 8 sublattices, and i and j correspond to a single site on each sublattice. The Hamiltonian can be rewritten in terms of the creation and annihilation operators of magnons by applying the Holstein Primakoff approximation. By defining the +b direction as the quantization axis, the creation and annihilation operators are introduced as $S_i^+ \sim \sqrt{2S}\hat{a}_i$, $S_i^- \sim \sqrt{2S}\hat{a}_i^\dagger$, $S_i^z \sim S - \hat{a}_i^\dagger\hat{a}_i$ if the magnetic moments is parallel to the +b direction. On the other hand, for the sublattice in which the magnetic moments is antiparallel to the +b direction, the creation and annihilation operators are introduced as $S_i^+ \sim \sqrt{2S}\hat{a}_i^\dagger$, $S_i^- \sim \sqrt{2S}\hat{a}_i$, $S_i^z \sim -S + \hat{a}_i^\dagger\hat{a}_i$. If magnetic moments on sublattice A are parallel or antiparallel to those of sublattice B, the Hamiltonian H_{AB} up to the second order of the creation and annihilation operators is given by,

$$H_{\mathrm{AB,parallel}} = -N_{\mathrm{AB}}S^2 + S\sum_{i\in \mathrm{A}}\sum_{j\in \mathrm{B}} J_{ij}(\hat{a}_i^\dagger\hat{a}_i + \hat{b}_j^\dagger\hat{b}_j + \hat{a}_i^\dagger\hat{b}_j + \hat{a}_i\hat{b}_j^\dagger), \tag{3}$$

$$H_{\mathrm{AB,antiparallel}} = N_{\mathrm{AB}}S^2 + S\sum_{i\in \mathrm{A}}\sum_{j\in \mathrm{B}} J_{ij}(-\hat{a}_i^\dagger\hat{a}_i - \hat{b}_j^\dagger\hat{b}_j + \hat{a}_i^\dagger\hat{b}_j^\dagger + \hat{a}_i\hat{b}_j), \tag{4}$$

respectively. S and N_{AB} represent a spin angular momentum and the number of the total bonds between the two sublattices, respectively. After the Fourier transformation, the Hamiltonian becomes

$$H_{\mathrm{AB,parallel}} = -N_{\mathrm{AB}}S^2 + S\sum_{\mathbf{k}}\{z_{\mathrm{AB}}(\hat{a}_{\mathbf{k}}^\dagger\hat{a}_{\mathbf{k}} + \hat{b}_{\mathbf{k}}^\dagger\hat{b}_{\mathbf{k}}) + \Gamma^*_{\mathrm{AB},\mathbf{k}}\hat{a}_{\mathbf{k}}^\dagger\hat{b}_{\mathbf{k}} + \Gamma_{\mathrm{AB},\mathbf{k}}\hat{a}_{\mathbf{k}}\hat{b}_{\mathbf{k}}^\dagger\}, \tag{5}$$

$$H_{\mathrm{AB,antiparallel}} = N_{\mathrm{AB}}S^2 + S\sum_{\mathbf{k}}\{-z_{AB}(\hat{a}_{\mathbf{k}}^\dagger\hat{a}_{\mathbf{k}} + \hat{b}_{\mathbf{k}}^\dagger\hat{b}_{\mathbf{k}}) + \Gamma^*_{\mathrm{AB},\mathbf{k}}\hat{a}_{\mathbf{k}}^\dagger\hat{b}_{-\mathbf{k}}^\dagger + \Gamma_{\mathrm{AB},\mathbf{k}}\hat{a}_{\mathbf{k}}\hat{b}_{-\mathbf{k}}\}, \tag{6}$$

where z_{AB} represent the number of bonds per a unit cell between the two sublattices. Γ_{AB} is the Fourier sum of the exchange coupling among the bonds connecting the two sublattices,

$$\Gamma_{\mathrm{AB},\mathbf{k}} = \sum_{\langle i\in \mathrm{A}, j\in \mathrm{B}\rangle} J_{ij}e^{-i\mathbf{k}\cdot(\mathbf{r}_j - \mathbf{r}_i)}. \tag{7}$$

The Bogoliubov-de Gennes Hamiltonian of $Fe_2Se_2O_7$ is constructed from adding all H_{AB} among the pair of the 8 sublattices.

A quadratic form is useful to represent all the terms included in the Hamiltonian. It is represented by a 16 × 16 matrix,

$$H = (\mathrm{const}) + \frac{1}{2}S\sum_{\mathbf{k}} t_{\mathbf{k}}^{\dagger} H_{\mathbf{k}} t_{\mathbf{k}} \tag{8}$$

$$H_{\mathbf{k}} = \begin{pmatrix} A_{\mathbf{k}} & 0 & B_{\mathbf{k}} & C_{\mathbf{k}} \\ 0 & A'_{\mathbf{k}} & C^{*}_{\mathbf{k}} & B'_{\mathbf{k}} \\ B_{\mathbf{k}} & C_{\mathbf{k}} & A_{\mathbf{k}} & 0 \\ C^{*}_{\mathbf{k}} & B'_{\mathbf{k}} & 0 & A'_{\mathbf{k}} \end{pmatrix}, \tag{9}$$

$$t_{\mathbf{k}} = {}^{t}(\hat{a}_{1\mathbf{k}}, \hat{a}_{2\mathbf{k}}, \cdots \hat{a}_{8\mathbf{k}}, \hat{a}^{\dagger}_{1-\mathbf{k}}, \hat{a}^{\dagger}_{2-\mathbf{k}}, \cdots \hat{a}^{\dagger}_{8-\mathbf{k}}), \tag{10}$$

$$A_{\mathbf{k}} = \begin{pmatrix} -2J_{\mathrm{vb1}} + 2J_{\mathrm{bb}} & 0 & J_{\mathrm{vb1}}\gamma_{31,\mathbf{k}} & J_{\mathrm{vb1}}\gamma_{41,\mathbf{k}} \\ 0 & 2J_{\mathrm{vb2}} + 2J_{\mathrm{bb}} & 0 & 0 \\ J_{\mathrm{vb1}}\gamma_{13,\mathbf{k}} & 0 & -J_{\mathrm{vb1}} + J_{\mathrm{vv}} + J_{\mathrm{vb2}} & 0 \\ J_{\mathrm{vb1}}\gamma_{14,\mathbf{k}} & 0 & 0 & -J_{\mathrm{vb1}} + J_{\mathrm{vv}} + J_{\mathrm{vb2}} \end{pmatrix} \tag{11}$$

$$A'_{\mathbf{k}} = \begin{pmatrix} -2J_{\mathrm{vb1}} + 2J_{\mathrm{bb}} & 0 & J_{\mathrm{vb1}}\gamma_{75,\mathbf{k}} & J_{\mathrm{vb1}}\gamma_{85,\mathbf{k}} \\ 0 & 2J_{\mathrm{vb2}} + 2J_{\mathrm{bb}} & 0 & 0 \\ J_{\mathrm{vb1}}\gamma_{57,\mathbf{k}} & 0 & -J_{\mathrm{vb1}} + J_{\mathrm{vv}} + J_{\mathrm{vb2}} & 0 \\ J_{\mathrm{vb1}}\gamma_{58,\mathbf{k}} & 0 & 0 & -J_{\mathrm{vb1}} + J_{\mathrm{vv}} + J_{\mathrm{vb2}} \end{pmatrix} \tag{12}$$

$$B_{\mathbf{k}} = \begin{pmatrix} 0 & J_{\mathrm{bb}}\gamma_{21,\mathbf{k}} & 0 & 0 \\ J_{\mathrm{bb}}\gamma_{12,\mathbf{k}} & 0 & J_{\mathrm{vb2}}\gamma_{32,\mathbf{k}} & J_{\mathrm{vb2}}\gamma_{42,\mathbf{k}} \\ 0 & J_{\mathrm{vb2}}\gamma_{23,\mathbf{k}} & 0 & 0 \\ 0 & J_{\mathrm{vb2}}\gamma_{24,\mathbf{k}} & 0 & 0 \end{pmatrix} \tag{13}$$

$$B'_{\mathbf{k}} = \begin{pmatrix} 0 & J_{\mathrm{bb}}\gamma_{65,\mathbf{k}} & 0 & 0 \\ J_{\mathrm{bb}}\gamma_{56,\mathbf{k}} & 0 & J_{\mathrm{vb2}}\gamma_{76,\mathbf{k}} & J_{\mathrm{vb2}}\gamma_{86,\mathbf{k}} \\ 0 & J_{\mathrm{vb2}}\gamma_{67,\mathbf{k}} & 0 & 0 \\ 0 & J_{\mathrm{vb2}}\gamma_{68,\mathbf{k}} & 0 & 0 \end{pmatrix} \tag{14}$$

$$C_{\mathbf{k}} = \begin{pmatrix} 0 & 0 & 0 & 0 \\ 0 & 0 & 0 & 0 \\ 0 & 0 & J_{\mathrm{vv}}\gamma_{73,\mathbf{k}} & 0 \\ 0 & 0 & 0 & J_{\mathrm{vv}}\gamma_{84,\mathbf{k}} \end{pmatrix}, \tag{15}$$

where

$$\gamma_{\mathrm{AB},\mathbf{k}} = \sum_{\langle i \in \mathrm{A}, j \in \mathrm{B} \rangle} e^{-i\mathbf{k}\cdot(\mathbf{r}_j - \mathbf{r}_i)}. \tag{16}$$

The matrix is paraunitary due to commutation relations of a bosonic operator. Thus, the dispersion relation can be obtained from diagonalizing the $\Sigma H_{\mathbf{k}}$, where Σ is a 16 × 16 matrix defined by a 8 × 8 identity matrix I,

$$\Sigma = \begin{pmatrix} I & 0 \\ 0 & -I \end{pmatrix}. \tag{17}$$

References

1. Sobolev, A. V. *et al.* Magnetic hyperfine interactions in a sawtooth chain iron oxoselenite fe2o(seo3)2: Experimental and theoretical investigation. *J. Alloy. Compd.* **822**, 153549, DOI: https://doi.org/10.1016/j.jallcom.2019.153549 (2020).

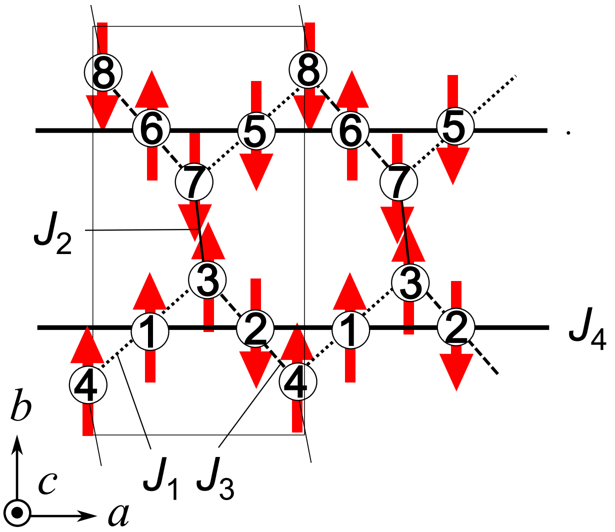

Figure S4. Schematic model of the antiferromagnetic structure and exchange constants used to calculate the dispersion relations.
